# Mesenteric cystic lymphangioma in adults: a rare entity presenting as acute abdomen - a report of two cases

**DOI:** 10.4322/acr.2024.470

**Published:** 2024-02-08

**Authors:** Tanvi Jha, Monika Sharma, Arvind Ahuja

**Affiliations:** 1 Atal Bihari Vajpayee Institute of Medical Sciences and Dr. Ram Manohar Lohia Hospital, Department of Pathology, New Dehli, India

**Keywords:** Abdomen, Acute, Adult, Lymphangioma, Mesentery, Lymphatics

## Abstract

Lymphangiomas are rare benign tumors that mainly involve the head and neck region in pediatric patients. Lymphangiomas of the small bowel mesentery in adults are rarer. We present two cases of mesenteric lymphangioma with acute abdominal pain on presentation. Case 1: A 38-year-old female presented with abdominal pain, vomiting, fever, and difficult evacuation. On abdominal examination, she had an ill-defined, tender lump, and radiological findings raised a possibility of perforation peritonitis. Thus, exploratory laparotomy was planned. Per-operatively, a mesenteric mass was found, which, on histopathological evaluation, was found to be a mesenteric lymphangioma involving the bowel. Case 2: A 27-year-old male presented with abdominal pain and difficult evacuation. Radiological evaluation revealed a multilobulated lesion involving the mesentery and with differential diagnoses of mesenteric fibromatoses and inflammatory pseudotumor. Histopathological assessment of the resected mass revealed a lymphangioma that was limited to the mesentery. Owing to their rarity and non-specific presentation, mesenteric lymphangiomas are often misdiagnosed on clinical examination and imaging. Thus, histopathological examination is the gold standard to reach a definitive diagnosis.

## INTRODUCTION

Lymphangiomas are rare benign tumors characterized by the proliferation of lymphatic vessels.^1^ They are typically seen in children, and adult cases are rare. They present mainly in the head and neck region and chiefly affect the skin and subcutaneous tissues.^2^ Less than 1% of cases occur in the mesentery and retro-peritoneum.^3^

Lymphangiomas occur due to congenital malformation of the lymphatic vessel or secondary to pre-existing conditions leading to lymphatic obstruction.^3^ Depending on the size of the dilated lymphatic spaces, they are of three histologic types: capillary (simple), cavernous and cystic.^1^ Most mesenteric lymphangiomas are asymptomatic or present with chronic abdominal pain when they are significantly enlarged. The presence of acute symptoms is rare and is usually associated with large lesions with complications such as obstruction, infarction, and perforation.^4^ These lesions may also infiltrate the surrounding tissues; timely detection and adequate surgical resection are necessary to prevent these complications and avoid recurrence.^5^ We, thus, present two cases of adult mesenteric lymphangioma, which presented with acute abdominal pain.

## CASE REPORT

### Case One

A 52-year-old female patient presented to the emergency room with a 7-day history of progressive colicky abdominal pain, vomiting, fever, chills, rigors, and an inability to evacuate. On abdominal examination, abdomen was grossly distended with localized rebound tenderness. An ill-defined, tender lump involving the right iliac fossa, right flank, umbilical, and hypo-gastric region was palpated.

**Figure 1:**
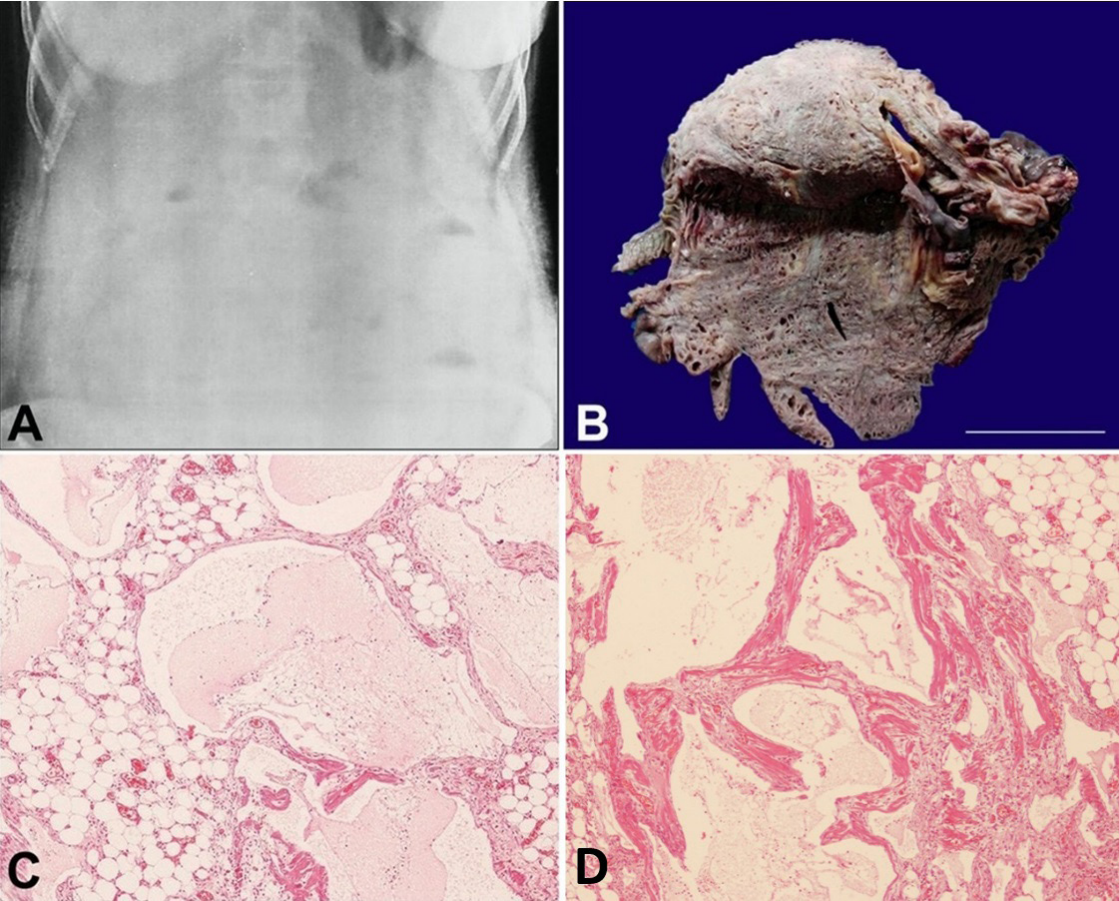
**A -** A plain abdominal radiograph revealing ground glass appearance with multiple air fluid levels in the abdomen; **B -** Gross specimen of small intestine with large cystic mesenteric mass encroaching the serosal surface of ileum and comprising of cysts containing milky fluid. (scale bar=10 cm); **C** and **D -** Photomicrographs of the surgical specimen; **C -** Variable sized dilated lymphatic channels lined by flattened endothelium within the mesenteric mass; and **D -** in the serosa (H&E, 400x).

The initial abdominal radiograph showed a ground glass appearance with multiple air-fluid levels (Figure 1A). A multiseptated organized collection filling the peritoneal cavity with dilated bowel loops showing sluggish peristalsis was noted on ultrasonography (USG). Thus, the pre-operative diagnosis included peritonitis secondary to perforation. An emergency exploratory laparotomy was, therefore, performed, and a lobulated mesenteric mass of approximately 30 cm in length encasing the ileum was found, which was resected. No perforation was identified intra-operatively.

On gross examination, the surgical specimen consisted of a 30 cm segment of the small intestine, along with a large, lobulated, soft to cystic mesenteric mass measuring 30×17×8 cm, encroaching the serosal surface of the ileum (Figure 1B). The cysts ranged from 0.1 to 0.7 cm in size and contained a milky or serous fluid.

**Figure 2:**
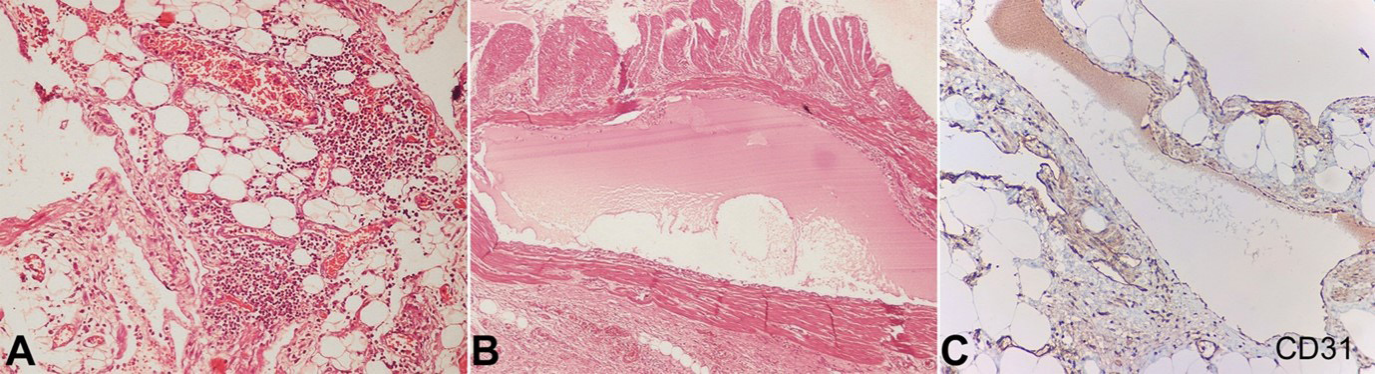
**A** and **B -** Photomicrographs of the surgical specimen showing mature adipose tissue interspersed with variable-sized, dilated lymphatic channels within the mucosa, submucosa, and muscularis propria (H&E, 400x); **C -** CD31 positive lymphatic channels in the mesenteric mass (CD31, 400x).

On microscopic examination, the mesenteric mass showed variable-sized dilated lymphatic channels (Figure 1CD). Sections from the intestine revealed similar dilated lymphatics within the serosa, muscularis propria, submucosa, and mucosa (Figures 2AB). These lymphatic channels demonstrated positivity with D2-40 and CD 31 (Figure 2C) by immunohistochemistry (IHC). Thus, a diagnosis of mesenteric lymphangioma with small bowel involvement was made.

### Case Two

**Figure 3:**
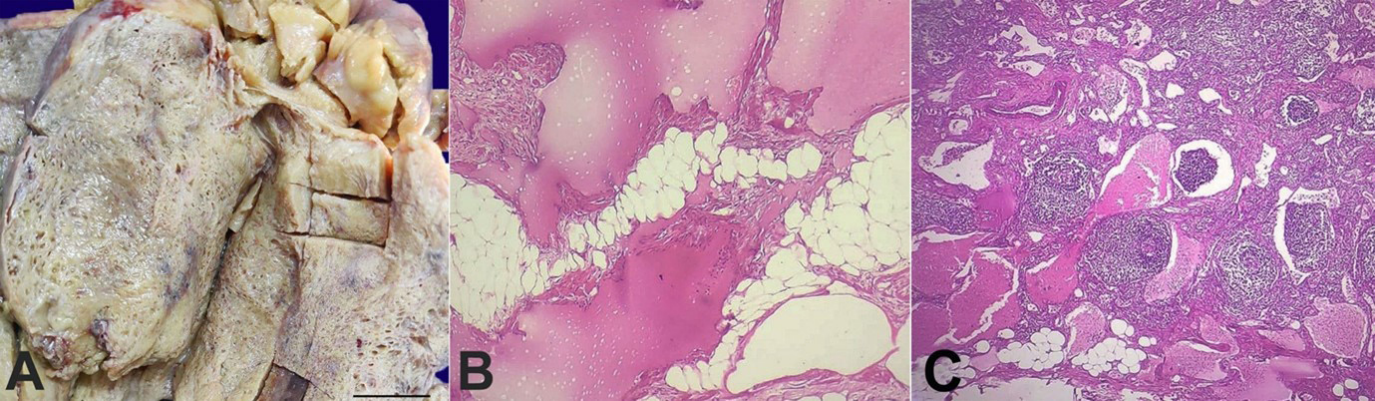
**A -** Gross specimen of large mesenteric mass with multiple tiny cysts and areas of vascular congestion. (scale bar= 4 cm); **B -** Variable-sized, thin-walled, dilated lymphatic channels lined by flattened endothelium (H&E, 400x); **C -** Dilated lymphatic channels with focal surrounding lymphoid aggregates (H&E, 400x).

A 27-year-old male presented to the emergency with a 2-day history of abdominal pain and an inability to evacuate. On abdominal examination, there was rigidity, rebound tenderness, and an ill-defined lump in the umbilical region. USG and non-contrast computed tomography revealed a multilobulated lesion involving the mesentery. The radiological differential diagnoses considered were mesenteric fibromatosis and inflammatory pseudotumor. The patient underwent surgery, and a 25 cm segment of the small intestine was resected, along with a large, circumscribed, soft to cystic mesenteric mass measuring 19×14×13 cm (Figure 3A). Serial sectioning of the mass revealed spongy tissue with multiple tiny cystic areas ranging in size from 0.1 to 0.4 cm, filled with serous fluid.

**Figure 4:**
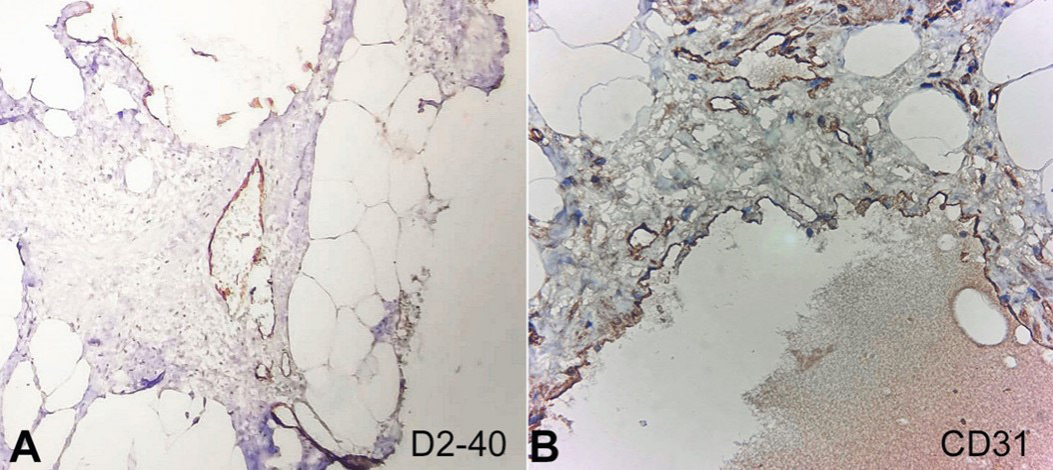
Photomicrographs of surgical sections stained with immunohistochemistry. **A -** D2-40 positive endothelial cells lining the lymphatic channels (200x); **B -** CD31 positive endothelial cells lining the lymphatic channels (200x).

Microscopically, multiple sections demonstrated mature adipose tissue interspersed with numerous thin-walled muscular, dilated lymphatic channels lined by flattened endothelium (Figure 3B). Focal surrounding lymphoid aggregates were noted (Figure 3C). Sections examined from the bowel were unremarkable. On IHC, D2-40 (podoplanin) and CD 31 were positive in these channels, confirming the lymphatic origin of the lesion (Figure 4AB). A final diagnosis of mesenteric lymphangioma (limited to the mesentery) was, thus rendered.

## DISCUSSION

Lymphangiomas comprise numerous thin-walled lymphatic spaces usually seen in the head, neck, and axillary regions in the first year of life. Other rare locations include the abdominal and mediastinal cavities.^1,3^ Small-bowel mesentery is a rare location, particularly in adults.^2^ However, amongst the reported cases, the distal ileal mesenteric location, as also seen in our cases, is the most common site in the gastrointestinal tract.^6^

Lymphangiomas occur due to congenital malformation of lymphatic vessels, leading to the sequestration of lymphatic vessels during the embryonic period.^3,7^ However, it has also been suggested that pre-existing inflammation, abdominal trauma, surgery, radiation, or lymphatic obstruction may result in their formation.^1,8,9^

Mesenteric lymphangiomas are usually asymptomatic and may be detected incidentally during surgical procedures for other conditions.^10^ Adult cases, like the two cases presented herein, may rarely be associated with acute abdominal symptoms. These cases are usually large, and symptoms may be due to associated mass effects.^4^ Volvulus is a common manifestation of small bowel mesenteric lymphangiomas.^6^

Chen et al.^5^ reported 6 cases of mesenteric lymphangiomas of the small intestine. Except for one, all of their cases presented with acute symptoms similar to our patients. However, the largest mass they reported measured 11×4 cm. Aprea et al.^11^ also reported 5 cases of mesenteric lymphangiomas of the small intestine, which ranged in size from 5 cm to 18 cm. Unlike our cases, all of these patients presented with chronic abdominal pain, except one, who was asymptomatic and was diagnosed incidentally. Others have also reported cases presenting with chronic symptoms and with masses smaller than those seen in our patients.^12,13^

The management of mesenteric lymphangiomas is surgical resection.^5^ In both of our cases, surgical intervention was necessary to address the patients' acute symptoms and establish a definitive diagnosis. Resection may vary in extent, depending on the lesion’s size and location.^14^ Surgical resection of mass in toto is curative and associated with a good prognosis. However, the risk of invasion of mesenteric lymphangiomas into adjacent structures is of critical consideration in these cases. These lesions can infiltrate surrounding tissues, leading to complications such as bowel obstruction, perforation, and peritonitis.^11^ Incomplete resection is associated with a risk of recurrence. The recurrence rate for mesenteric lymphangiomas is high, and thus, long-term follow-up is advisable.^14^ Additionally, the outcomes may vary depending on factors such as the lesion’s extension, histopathological characteristics, and complications at the time of the diagnosis.^5,12^ Our cases underwent surgical excision and are currently under routine follow-up.

Therefore, while mesenteric lymphangiomas are usually asymptomatic and, thus, remain a diagnostic quandary, timely detection and early removal promise favorable outcomes.

## CONCLUSION

Our patients were both adults who presented with non-specific symptoms of acute abdominal pain and obstruction owing to the complications associated with their large abdominal mass. Mesenteric lymphangiomas are exceedingly rare in adults and can present with various clinical and histopathological features. Though the prognosis is favorable, there is potential for recurrence; hence, long-term follow-up may be required.
